# Environmental Management in Small and Medium-Sized Companies: An Analysis from the Perspective of the Theory of Planned Behavior

**DOI:** 10.1371/journal.pone.0088504

**Published:** 2014-02-12

**Authors:** Agustín J. Sánchez-Medina, Leonardo Romero-Quintero, Silvia Sosa-Cabrera

**Affiliations:** 1 Department of Economics and Management, University of Las Palmas de Gran Canaria, The Canary Islands, Spain; 2 University Institute of Cybernetic Science and Technology, University of Las Palmas de Gran Canaria, The Canary Islands, Spain; Universita' del Piemonte Orientale, Italy

## Abstract

In the business context, concern for the environment began to develop when pressure from the public administration and environmental awareness groups raised the specific requirements for companies. The Theory of Planned Behavior considers that people's conduct is determined by the intention of carrying out a certain behavior. Thus, the individual's intent is determined by three factors related to the desired outcome of the behavior: the Personal Attitude toward the Results, the Perceived Social Norms, and the Perceived Behavioral Control over the action. Therefore, the objectives of this paper are to clarify the attitudes of the managers of Canarian small and medium-sized companies about taking environmental measures, and try to demonstrate whether there is a relationship between the proposed factors and the intention to take these measures.

## Introduction

During the past decade, the globalization of economic activity has led to an intensification of competition on a worldwide scale. This situation is causing companies that do not adapt to this new scenario to see reduced profit margins. They are then forced to devise strategies to achieve competitive advantages that would allow them to recover or improve their profitability. Therefore, the study of the determinants of business competitiveness becomes a matter of great importance. The key is to know what the origins of the competitive advantages in a market are, and what to do to maintain and/or improve these advantages [1]. Until well into the eighties, knowledge of the environment meant that the main areas of interest in the study of business competiveness were the sectorial analysis and competition, focusing on these non controllable factors of the company [2]–[6]. However, since the end of the same decade, many studies have highlighted the need to examine not only the markets, but also the behavior of the organization [7]–[11].

On the other hand, growing concern about environmental deterioration has resulted in pressure for companies to incorporate more respectful behavior toward the environment, and this can be used as a strategic issue for making businesses competitive. In this context, small and medium-sized enterprises (hereinafter SMEs), which provide the economic and business structure of many territories, play an important role. In fact, in the European Union, SMEs account for 99% of the existing companies [12]. Therefore, their contribution to the creation of value and employment, as well as their environmental impact, can be considerable. Although it is clear that the contribution of only one SME to the sustainable development of a region is relatively small, considered as a group, they have a significant influence on the quality of the development of a particular geographical area, even exceeding that of the larger companies on occasion. In this respect, the greater the presence of SMEs in the economy or in a territory, the greater their influence is on the level of sustainable economic development [13]–[15].

The academic field has found it difficult to establish a precise and consensual definition of the concept of an SME. Thus, the specialized literature provides numerous criteria to characterize SMEs, such as the number of employees, turnover, total assets, etc. [16]–[20]. To the disadvantages of this lack of consensus, one must add the existing differences among the characteristics of SMEs, depending on the sector in which they operate. Due to these discrepancies, researchers have applied different definitions in their studies.

All of this has led governments and organizations from different countries to establish different criteria for defining what types of companies can be considered SMEs [21]. Thus, since 2005, the Commission of the European Communities states that for a company to be considered an SME, the maximum limits are as follows: 250 employees, 50 million Euros turnover, and 43 million Euros in assets [22]. Spain's General Accounting Plan is the legal text that regulates the accounting within companies. It specifically indicates that “*[…] 1. All companies will be able to apply this General SME Accounting Plan, whatever their juridical form (individual or corporate), which at the end of two consecutive fiscal years must meet at least two of the following criteria: a) The total assets must not exceed 2,850,000 Euros; b) The net amount of their annual turnover must not exceed 5,700,000 Euros; c) The average number of employees during the fiscal year must not exceed 50.*” [23].

The purpose of this paper is to examine the perceptions of managers of SMEs, as defined in the criteria established in the Spanish regulations, about the need to implement environmental measures within their companies. Thus, based on the Theory of Planned Behavior [24], in which intent is one of the best predictors of people's planned behavior, it is interesting to determine whether an SME manager intends to carry out measures to improve the company's environmental behavior. Therefore, the overall objective of this study is to test the ability of the factors proposed in Ajzen's model to predict the intention of undertaking environmental measures in SMEs. An empirical analysis was carried out in companies located in Gran Canaria (Canary Islands – Spain) whose high energy consumption or high waste production could cause major environmental problems in their daily management.

## Company and Environment

Although it is true that for a long time the economic and productive activities of mankind have had a strong relationship with the environment, only in recent years has society become concerned about the negative effects this relationship might have on the environment.

Until a few decades ago, from an analytical perspective, the business activity was considered a closed system in itself, where economic agents, consumers or producers behaved in rational ways, seeking to maximize their welfare, without taking into account the impact of their actions on the social and physical environment. While in the past there was no need to worry about possible environmental damages because nature itself solved the majority of the problems arising from production processes, distribution and consumption through recycling and biological processes [25], the situation has changed significantly. Recent social and institutional concerns about environmental deterioration have produced pressure on companies to introduce more respectful behavior toward their natural environment.

**Figure 1:**
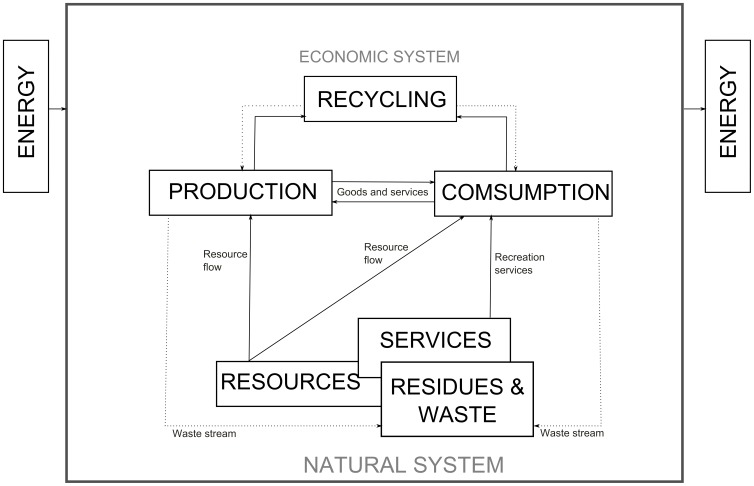
Environment and Economy. Source: Adapted from Ruesga and Durán [27] and Del Brio and Junquera [28].

Nevertheless, before introducing these new environmental perspectives into the business, one should be aware that the environment has three significant functions [26]. First, it is the fundamental source of resources, as the environment is a supplier of the natural inputs for the production process. Second, nature provides recreational services related to enjoying the environment, such as scenic beauty, clean air, etc. And finally, the natural environment assimilates the waste and residues generated in this production and consumption. However, it must be acknowledged that only when the amount of waste discharged into the environment is within the limits set by its assimilative capacity can nature maintain its role as a repository of this waste (see Figure 1).

Traditionally, businesses and the environment have been, and in certain aspects continue to be, conflicting elements: business is a threat to the environment, and environmental concern presents obstacles to business development and job creation. In recent years, however, this opposition has been overcome, while the concept of sustainable development has been imposed. As indicated by Ruesga and Duran [27], businesses and the environment are destined to understand each other: the company plays a leading role in investigating and contributing technological solutions for environmental problems; and for the company, the environment constitutes a rapidly expanding market, a business opportunity, and an opportunity to create employment.

All business organizations, regardless of their size, activity or scope, generate environmental problems to a greater or lesser degree, and they must meet the challenge of complying with the requirements of the natural environment in which they operate. These requirements are demonstrated in the form of pressure from its stakeholders - any group or individual who can affect or be affected by the achievement of the organization's objectives or its actions [29].

**Figure 2:**
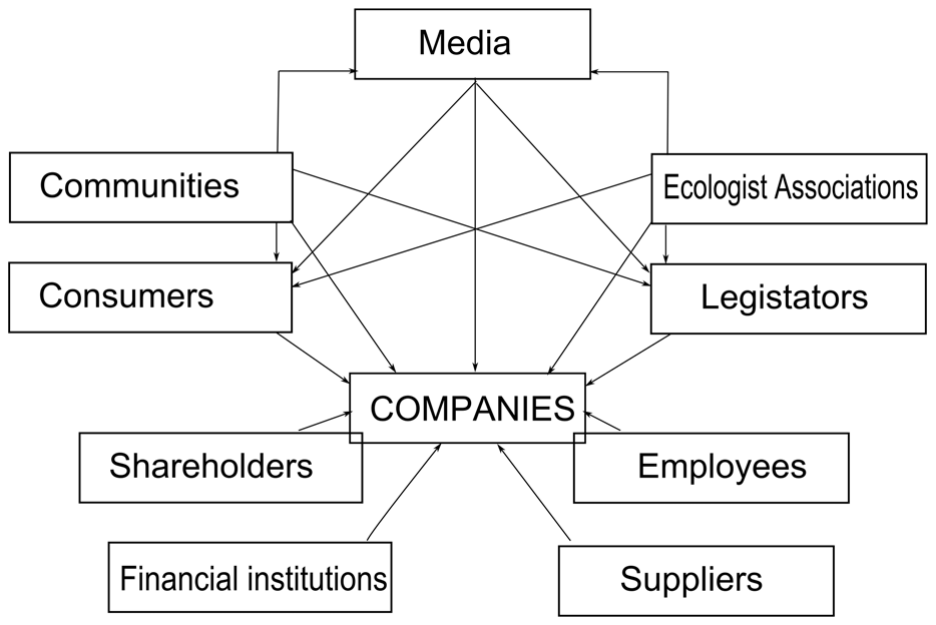
Environmental Stakeholders. Source: Garcés [36].

The literature includes empirical research confirming the stakeholder demands that determine the companies' environmental strategies [30]–[35]. Figure 2 summarizes the major stakeholders who have the ability to influence companies' environmental policies.

It seems clear that the different environmental obligations will increase in the future, with more demanding legislation and increasingly strong pressure from society and the market. Therefore, companies will need to adapt to this situation by adopting certain ecological principles. This adaptation process will generally require a transformation of the companies, their products, their production systems, and their management practices [37], which will translate into a reduction in companies' impact on the natural environment. In short, the environment has become a strategic issue for businesses [38], [39], and it requires constant attention and a suitable integration of all its aspects in the company's strategy. As a result, company managers must take steps to bring about environmental improvements.

## Intention Model to Undertake Environmental Measures

Research in social psychology has widely used Fishbein and Ajzen's theory of rational action [40] to investigate the relationship between motivation and a wide variety of behaviors [41], [42], proving successful in many cases [43].

The fact that not all individuals behave in the same way in similar situations implies that their behavior is influenced by internal variables. Managers are no exception; therefore, they do not all have the same willingness to undertake certain actions to pursue a specific purpose. Proof of this is that the individual's psychological attitudes form a key part of the research on the entrepreneurial phenomenon [44], both within and outside the company.

This study will focus specifically on environmental initiatives within a company. It is assumed that such actions can be considered planned behavior. As can be deduced from the academic literature on psychology, intentions have been shown to be the best predictor of planned behavior. Thus, according to Ajzen [24], intentions help to understand the act itself, leading to the question of whether the factors that make up the intentions model encourage the act of carrying out environmental measures. Managers' intentions are of great interest in this sense, as they correspond to their state of mind and focus their attention on this objective.

The intention begins the process of starting an action. Therefore, models that explain the cognitive process that leads managers to act, based on these intentions, are presented as an alternative to stimulus-response models in attempting to understand their behavior. Moreover, social psychology offers intention models that can be used to explain or predict social and managerial behaviors. These models provide a theoretical framework that specifically shows the nature of the process underlying the intentional behavior. According to Krueger *et al.* [45], intent-based models have been applied to explain the manager's behavior in several studies [46], [47].

Some authors have based their papers on the search for the existence of certain personality traits associated with entrepreneurial activity [48]. Others have focused on demonstrating the importance of demographic characteristics, such as age, sex, place of origin, religion, education, work experience etc. [49]. These two types of analysis have led to the identification of significant relationships between certain traits or demographic characteristics and the performance of managerial behaviors. Nevertheless, their predictive ability has been very limited [50]. With regard to the theoretical aspect, many authors have criticized both the methodological and conceptual problems presented [24], [51], [52]. Along the lines of the studies by Shapero and Sokol [51] on the Entrepreneurial Event model and Fishbein and Ajzen [40] on the Theory of Reasoned Action, Ajzen [24] contributes his Theory of Planned Behavior. The intention of performing a behavior depends on the subject's attitude toward that behavior [24]. Therefore, it seems reasonable that if a manager has a favorable attitude toward performing a certain behavior, he/she is much more likely to do so. Thus, this approach based on individuals' attitudes is superior to others based on their traits or demographic aspects [45], [53].

**Figure 3:**
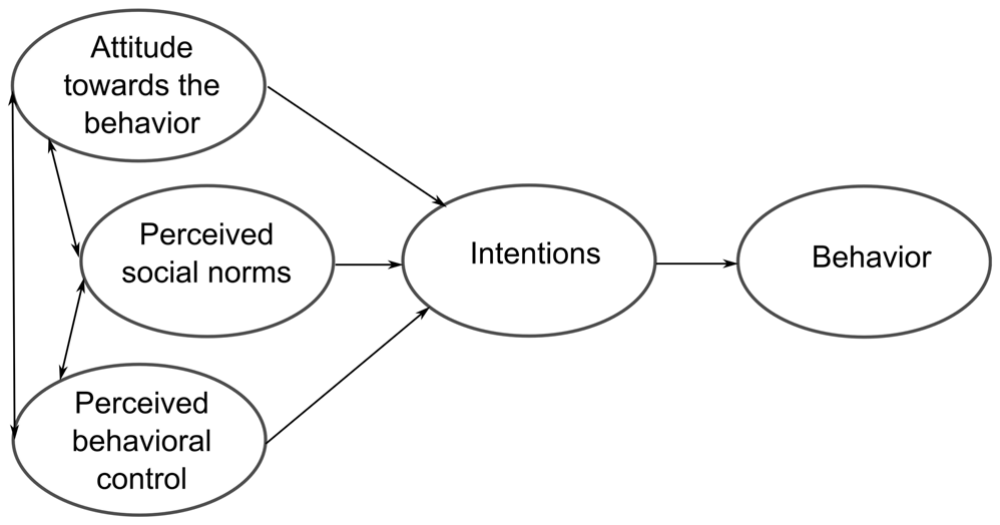
Ajzen's model of planned behavior. Source: Ajzen [24].

The Theory of Planned Behavior has been used in many fields, particularly in the managerial area. Many studies have linked this theory to the decision to create a firm [45], [46], [54]–[60]. Alternatively, this theory has also been applied to the study of pro-environmental behavior, focusing mainly on the attitudes of individuals and households toward recycling, their environmentally aware attitudes, and reducing pollution [61]–[69]. However, we found no research in the literature that applied the factors used in the Ajzen model to implementing environmental measures in SMEs. Therefore, important work remains to be done on this topic. The present paper attempts to contrast whether the factors proposed by Ajzen [24] in his theory influence the decisions made by SME managers about their company's environmental performance. Intentionality is said to be influenced by three aspects (see figure 3) whose relative importance is expected to vary for different situations and behaviors. Therefore, in this paper, we attempt to verify whether the social norms about taking environmental measures, the ability of managers to do so, and their attitudes toward these norms influence their intention of carrying them out. These decisions, as discussed throughout this paper, are becoming increasingly relevant for both companies and society in general.

**Figure 4:**
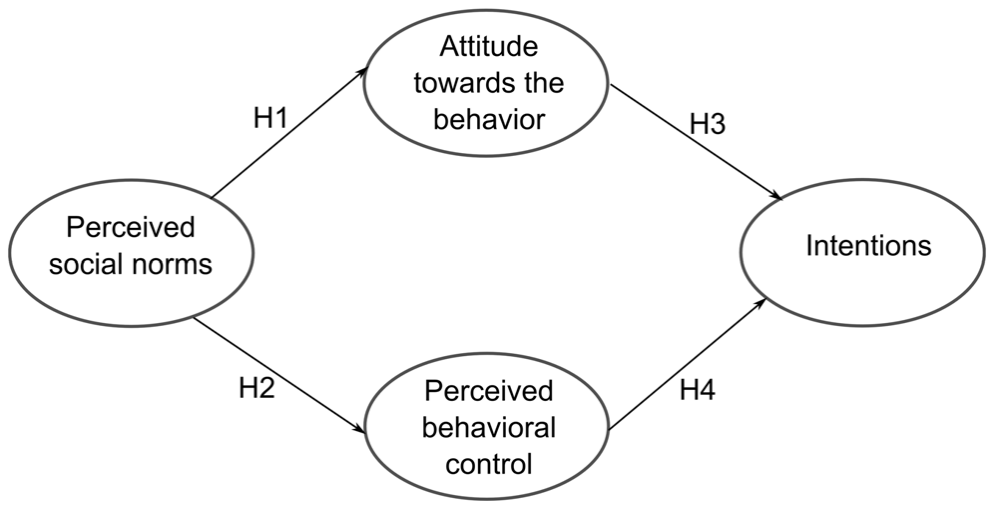
Sample of the conditioning intentionality Model.

In the context studied in this paper (see figure 4), the perceived social norm or the subjective norm can also help us to explain the intention of carrying out environmental measures. In particular, the idea of the subjective norm means that the personal perceptions of the company's important reference groups, who believe that the behavior in question must be carried out, could influence both the Attitude toward the Behavior and the Perceived Behavioral Control, all of which lead to the following hypothesis:

*H1: The Perceived Social Norms positively influence the Attitude toward the Behavior of undertaking environmental measures.*

*H2. The Perceived Social Norms positively influence the Perceived Behavioral Control for undertaking environmental measures.*

The attitude-intention relationship used in the Ajzen model [24] should also be present. Therefore, the intention of taking environmental actions should increase if the manager has a positive attitude toward taking these measures. This relationship is reflected in the following hypothesis:

*H3: The Attitude toward the Behavior positively influences the intention to undertake environmental measures.*

Finally, greater perceived control, defined as the perception of ease or difficulty of performing the behavior that the manager wants to perform, favors an increase in the intention of performing environmental actions. This hypothesis has also been extensively validated in studies that use the theory of Planned Behavior, and in the environmental context it translates into the following relationship:

*H4: The Perceived Behavioral Control positively influences the intention to undertake environmental measures.*

According to Liñán and Chen [47], the external variables only have a direct influence on the antecedents of intention. Therefore, in the present study the control variables are included to explain the Attitude toward the Behavior and the Perceived Behavioral Control. Two types of control variables have been used, those related to the demographic information of the business owner (gender, educational level and work experience) and those related to the organizational characteristics of the company (size and activity sector).

The demographic variables included in this study have commonly been used in the literature on intentionality [46], [47], [70]–[72]. Work experience has been included, for example, in studies like those by Liñán and Chen [47], Robinson et al. [53], Kibler [70], Nabi and Liñán [71]. Another variable related to the demographic characteristics of the business owner that has frequently been employed in this type of studies is the educational level [47], [73], [74].

On the other hand, given that social responsibility actions, among them environmental ones, are conditioned by characteristics of the company [75]–[79], two control variables were also included that have to do with these characteristics. The first was company size, measured by the number of employees. This variable is relevant because it has traditionally been positively associated with social performance [76], [77]; as companies grow, they focus more attention on the stakeholders and need to more effectively respond to their demands [78], [80]. The other variable considered was the activity sector to which the company belongs. It was included because, as stated by Waddock and Graves [76] and Graves and Waddock [81], it is relevant to control differences stemming from pertaining to a certain activity sector.

## Design and Research Methodology

It is necessary to emphasize that the managers who were chosen as the object of this study were in charge of small businesses located on the Island of Gran Canaria whose activity causes significant environmental problems. The decision to study SMEs is mainly due to the importance that these types of businesses have in the economy. Before the crisis of the 1970's, production and job creation were concentrated in large firms [82]. However, from that decade on, a change in tendency was detected that produced an increase in the importance of SMEs to national economies, as Loveman and Segenberger [83] and Schwalbach [84] confirmed in empirical studies. On the other hand, the competitiveness of these types of companies depends, fundamentally, on the capabilities of the manager or owner, on investments in intangible and technological equipment, and on their flexible innovation capacity [85]. Therefore, this paper focuses on the manager or owner and, more specifically, on the intention and the constraints of environmental measures.

In this research, the method used to obtain the necessary information to fulfill the stated objectives was a survey whose basic observation tool was a questionnaire [86]. The questionnaire was carried out by interviewers who contacted the managers directly. This procedure, although more costly than self-administered questionnaires, ensured that the answers were given by the desired people, and that the task was not given to someone else within the company. The same interviewers made contact with the managers and set up an appointment to complete the questionnaire. However, before proceeding with each questionnaire, an exploration study was carried out in order to obtain an estimate of the actual state of each SME and the managers' perceptions of the importance of intangibles in running their businesses.

Finally, it must be noted that a pretest was conducted on ten managers, which helped us to highlight the questions that would not be clearly understood or that could lead to confusion when responding. After the pretest was carried out, we made alterations in several questions in order to ensure that the respondents were able to fully understand them.

Questionnaire responses were obtained from 201 SMEs. Table 1 summarizes the data from the quantitative research undertaken. The sample error was 6.6%, with a confidence level of 95%. It should be pointed out that, as mentioned above, the chosen spectrum did not include all SMEs in Gran Canaria, but only those whose high energy consumption or high waste production could cause major environmental problems in their daily management. Thus, the companies studied were vehicle repair shops, carpentry businesses, bakeries and restaurants. This research paper has been examined by members of the Human Research Ethics Committee of the University of Las Palmas de Gran Canaria, who have met for this purpose, and a special authorization is not considered to be required.

**Table 1:** Research Factsheet.

Aspects of the Research	Description
Methodological procedure	Survey
Question Types	Categorised
Spectrum	SMEs with problematic environmental issues
Geographical scope	Gran Canaria
Data collection method	Questionnaire carried out by interviewers
Sample size	201 Companies
Confidence level	95% p = q 50%
Sample error	6.6%
Date of completion of the pretest	February 2012
Date of completion of the fieldwork	February 2012 to June 2012

From the total census of the above-mentioned companies, which was obtained through the SABI database (Bureau Van Dijk – Financial company information and business intelligence for companies in Spain and Portugal), a random sampling was conducted within each group to choose the organizations that participated in the study.

To measure the different constructs, a Likert-type scale was used. The point system followed this format: 1 (strongly disagree) to 7 (strongly agree), adapted from the Entrepreneurial Intention Questionnaire (EIQ) proposed by Liñán and Chen [47].

The control variables were measured in the following way. For gender, a dichotomous variable was used, where a value of 0 was assigned to men and 1 to women. For work experience, a four-level scale was used, with a value of 1 for less than five years of experience, 2 for between 5 and 10 years, 3 for between 11 and 15 years, and 4 for more than 15 years of experience. Educational level was measured on a five-level scale, with a value of 1 for primary studies or no studies, 2 for secondary education, 3 for vocational or technical education, 4 for a university degree, and 5 for a post-graduate degree.

For company size, a five-level scale was used where the following values were assigned according to the number of employees: 1 for less than 10; 2 for 10 to 24; 3 for 25 to 49; 4 for 50 to 99; and 5 for more than 100. Finally, in the activity sector, as six categories were established (food, automobile repair and maintenance, wood and metal industry, hospitality and restaurant, other small industries, and sales), 5 dichotomous indicators were used, that is, n-1 categories. Each of these dichotomous variables was associated with one of the first 5 categories mentioned previously, with these variables taking a value of 1 when the company was included in the sector linked to this variable and 0 in the opposite case. To represent the sixth category, all the indicators were given a value of 0.

The fieldwork was completed, and the data obtained were codified and set in table form. For this, the statistical program SPSS (Statistical Package for Social Sciences) for Windows version 19 was used. The Analysis of structural equations using the Partial Least Squares (PLS) technique was also applied to study the data. This methodology, which uses the algorithm of the Ordinary Least Square (OLS), was designed to reflect the theoretical and empirical aspects of the social qualities of the behavioral sciences, where there is generally sufficient theoretical support but little available information [87]. The PLS method is considered to be the most suitable when samples are relatively small, as in the case of this research [88]. This study specifically used SmartPls software version 02.00 [89].

The extent of common method bias was assessed with Harman's one-factor test, and it was performed by including all the items in a principal components factor analysis [90]. Evidence of common method bias exists when one factor accounts for most of the covariance. Thus, as suggested by Podsakoff and Organ [91], both the independent variables and dependent variables were included in the factor analysis. The factor analysis produced 5 factors, with none of them explaining the majority of the total variance.

The use of PLS requires two steps [92]. The first consists of the evaluation of the measurement model, in order to determine whether the relationship between the observed variables and the theoretical concepts or constructs being measured is correct. In order to carry out this analysis, the individual reliability of each item, the reliability of the construct, the Average Extracted Variance (AVE), and the discriminate validity of the indicators as measurements of the latent variables or constructs were evaluated. In order to analyze the reliability of the measurement scales, the Cronbach's alpha, among other statistics, was used. Its value ranges from zero to one, with the possibility of adopting negative values, which would imply that some of the items were measuring contrary elements. Thus, the closer the value of this statistic is to the item, the greater the internal consistency of the indicators on the valued scale [93].

The second step consists of the evaluation of the proposed model. The aim is to confirm the extent to which causal relationships specified by the proposed model are consistent with the data available. In this way, one can endeavor to test how much of the variance in the endogenous variables is explained by the predicted constructs. The measurement of the predictive power of a model is given the value R^2^ for the latent dependent variables.

The stability and validity of the estimates were examined using the *t-statistic* obtained by the bootstrap test with 500 subsamples. Finally, to verify the validity of the model, the Stone-Geisser test (Q^2^) was carried out. This test was used as a criterion to measure the predictive relevancy of the dependent constructs. In the case of Q^2^>0, the model has predictive relevancy, and in the opposite case, it does not.

## Results

The sample in this study consisted of 201 companies on the Island of Gran Canaria. These companies are SMEs that are susceptible to having major environmental problems. Those companies that were not included due to their characteristics had no significant environmental problems and, consequently, no need to implement environmental measures. As a result, footwear and textile companies and small bazaars were not included, but vehicle repair shops, bakeries, carpentry workshops etc., were included. As already noted above, the company selection was random, and the interviews were held with individuals in positions of responsibility within the companies.

In the sample,154 of the interviewees were males (76.6%), leaving a percentage of 23.4% of women. Regarding the educational level, 22.4% had finished primary studies or less, 25.6% had a university degree, and the rest had finished secondary or vocational education.

Regarding work experience, the majority of the business owners had a lot of experience. Thus, only 7.5% had less than 5 years of experience. As far as the legal set-up of the analyzed companies is concerned, approximately 60% of the companies were limited companies, followed by SA Corporations with 22.5%, and, finally, self-employed with only 17,3%.

Regarding the activity sector, 6 categories were established: food, automobile repair and maintenance, wood and metal industry, hospitality and restaurant, other small industries (printing, paint factories, industrial hygiene, etc.) and sales. The highest percentage of business owners surveyed belonged to the food sector (22.4%), and the lowest to the wood and metal industry (10.9%) (See Table 2 for more details).

**Table 2:** Description of the sample.

GENDER	Frequency	Percent	Valid Percent	STUDIES	Frequency	Percent	Valid Percent
Male	154	76.6%	76.6%	Primary or no studies	45	22.4%	23.4%
Female	47	23.4%	23.4%	Secondary	45	22.4%	22.6%
Total	201		100.0%	Vocational or technical education	53	26.4%	27.0%
				Degree	51	25.4%	26.0%
**EXPERIENCE**	**Frequency**	**Percent**	**Valid Percent**	Postgraduate degree	2	1.0%	1.0%
Less than 5 years	15	7.5%	7.8%	Total	196		100.0%
Between 5 and 10 years	40	19.9%	20.8%	Missing	5	2.5%	
Between 11 and 15 years	47	23.4%	24.5%				
More than 15 years	90	44.8%	46.9%	**ACTIVITY SECTOR**	**Frequency**	**Percent**	**Valid Percent**
Total	192		100.0%	Food	45	22.4%	23.4%
Missing	9	4.5%		Automobile	29	14.4%	15.1%
				Wood and metal	22	10.9%	11.5%
**SIZE**	**Frequency**	**Percent**	**Valid Percent**	Hospitality and restaurant	26	12.9%	13.5%
Less than 100 employees	111	55.2%	57.2%	Other small industries	32	15.9%	16.7%
Between 10 and 24 employees	53	26.4%	27.3%	Sales	38	18.9%	19.8%
Between 25 and 49 employees	14	7.0%	7.2%	Total	192		100.0%
Between 50 and 99 employees	6	3.0%	3.1%	Missing	9	4.5%	
More than 100 employees	10	5.0%	5.2%				
Total	194			**LEGAL set-up**	**Frequency**	**Percent**	**Valid Percent**
Missing	7	3.5%		Self-employed	33	16.4%	17.3%
				Limited companies	115	57.2%	60.2%
				SA Corporations	43	21.4%	22.5%
				Total	191		100.0%
				Missing	10		

### Analysis of the measurement model

To evaluate the measurement model, we must first note the individual reliability of each item. This procedure was carried out by examining the charges or simple correlations of the measurements or indicators with their respective constructs. According to Carmines and Zeller [94], in order to accept an indicator as part of a construct, it must have a value > = 0.707, implying that the variance shared between the construct and its indicators is greater than the error variance. However, other authors [92], [95] argue that it should not be so restrictive, and that indicators exceeding 0.65 should not be eliminated. As seen in Table 3, all indicators meet the conditions that exceed the value of 0.707.

**Table 3:** Outer model loadings and cross loadings.

	Attitude toward the Behavior	Intentions	Perceived Behavioral Control	Perceived Social Norms
**atb1**	**0.823**	0.385	0.293	0.383
**atb2**	**0.875**	0.414	0.225	0.298
**atb3**	**0.904**	0.397	0.252	0.376
**atb4**	**0.847**	0.472	0.213	0.309
**int1**	0.405	**0.851**	0.424	0.301
**int2**	0.523	**0.782**	0.423	0.382
**int3**	0.356	**0.826**	0.451	0.373
**int4**	0.376	**0.913**	0.450	0.410
**int5**	0.409	**0.934**	0.451	0.421
**pbc1**	0.210	0.339	**0.801**	0.354
**pbc2**	0.181	0.418	**0.885**	0.345
**pbc3**	0.298	0.444	**0.890**	0.479
**pbc4**	0.291	0.548	**0.899**	0.414
**psn1**	0.448	0.440	0.388	**0.777**
**psn2**	0.274	0.273	0.333	**0.841**
**psn3**	0.358	0.445	0.460	**0.820**
**psn4**	0.153	0.280	0.337	**0.758**
**psn5**	0.299	0.257	0.271	**0.769**
**psn6**	0.257	0.293	0.335	**0.741**

A second condition to bear in mind is the internal consistency, in order to assess the rigor with which the manifest variables are measuring the same latent variable. For this purpose, the composite reliability should be >0.7. As shown in Table 4, all cases exceed the value of 0.90. In the same table, it can also be seen that in each case Cronbach's alpha values superior to 0.87 are presented, which leads us to affirm that our constructs are reliable.

**Table 4:** Construct reliability, convergent validity and discriminant validity.

AVE	Composite Reliability	Cronbach's Alpha		Attitude toward the Behavior	Intentions	Perceived Behavioral Control	Perceived Social Norms
0.744	0.921	0.885	Attitude toward the Behavior	**0.862**			
0.745	0.936	0.913	Intentions	0.485	**0.863**		
0.756	0.925	0.893	Perceived Behavioral Control	0.287	0.511	**0.870**	
0.616	0.906	0.877	Perceived Social Norms	0.399	0.440	0.461	**0.785**

Diagonal elements (bold) are the square root of variance shared between the constructs and their measures (AVE).

Off-diagonal elements are the correlations among constructs.

For discriminant validity, the diagonal elements should be larger than the off-diagonal elements.

In the third step to evaluate the validity of the scale used, the Average Variance Extracted (AVE) was studied. Fornell and Larcker [96] recommend that the AVE be greater than 0.5, which means that over 50% of the construct variance is due to its indicators. As reflected in Table 3, this requirement is met in all the constructs used. The AVE exceeds 61% in the Perceived Social Norms, and values exceeding 74% can be seen in Perceived Behavioral Control, Intentions, and Attitude toward the Behavior.

Finally, we analyzed the discriminant validity, which tells us to what extent a model construct is different from other constructs that make up the model. One way to verify these circumstances is to demonstrate that the correlations between the constructs are lower than the square root of the AVE. Table 3 shows the correlation matrix of constructs as having replaced the value of the correlation in the diagonal by the square root of the AVE. As the diagonal values are the highest value in each row and column, we can affirm the existence of discriminant validity.

As all the previous tests were positive, it is now possible to state that the measurement model is valid and reliable. In the next paragraph, the evaluation of the proposed model under study will be examined.

### Proposed model Evaluation

**Figure 5:**
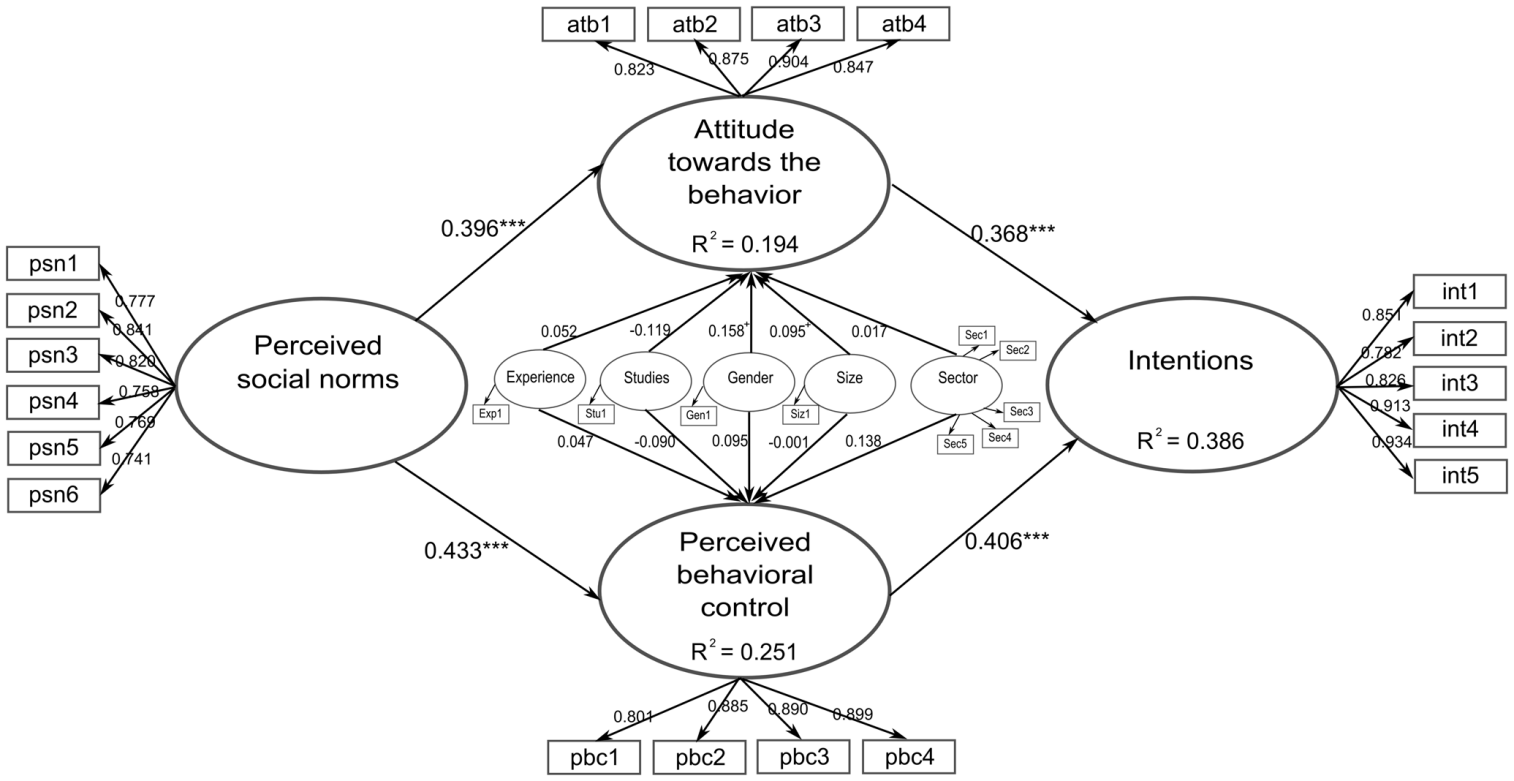
Indicator Charges of different constructs.

Once the validity of the measurement model had been studied, the causal relationship proposed in the model was evaluated. Thus, the quantity of the endogenous variances explained by the predicted constructs can be observed. The measurement of a model's predictive power is the value of R^2^ for the latent dependent variables. Figure 5 shows that the value of R^2^ for Intention is 0.386, indicating that 38% of the variance in this construct is explained by the model. In other words, pending the verification of the validity of this relationship, which will be carried out next, the findings show that almost 38% of the variance of the variable “intention to undertake environmental measures” is determined by the Attitude toward the Behavior and the Perceived Behavioral Control. Moreover, and although they are secondary results, it is also possible to state that the construct variance in the Perceived Behavioral Control is explained by 19.99% of the Perceived Social Norms. Finally, 15.77% of the Attitude toward the Behavior variance is derived from the Perceived Social Norms (see Table 5).

**Table 5:** Effects on endogenous variables.

	R^2^	Q^2^	Direct effect	Correlation	Variance explained
Attitude toward the Behavior	0.194	0.122			
H1: Perceived Social Norms			0.396	0.399	15.77%
Sector			0.017	0.120	0.21%
Size			0.095	0.068	0.64%
Studies			−0.119	−0.099	1.19%
Experience			0.052	0.114	0.59%
Gender			0.158	0.064	1.00%
Perceived Behavioral Control	0.251	0.188			
H2: Perceived Social Norms			0.433	0.461	19.99%
Sector			0.138	0.230	3.19%
Size			−0.001	−0.016	0.00%
Studies			−0.090	−0.127	1.15%
Experience			0.047	0.139	0.66%
Gender			0.095	0.015	0.14%
Intentions	0.386	0.273			
H3: Attitude toward the Behavior			0.368	0.485	17.85%
H4: Perceived Behavioral Control			0.406	0.511	20.74%

Table 6 shows the path values of the various relationships proposed in the study. To evaluate the validity of these relationships, we used the Bootstrap technique, which provided the standard deviation and the *t-statistic*. This way, the stability of the estimates was examined using a t-Student distribution with a line obtained from the Bootstrap Test with 5000 subsamples [97]. If we observe the values obtained, in every given *path* the value exceeds 3.106, which was established in the *t-statistic* as having a significance level of 0.001. On the other hand, some authors have proposed that, in addition to the analyses mentioned, the confidence interval of the paths should be studied. Thus, “If a confidence interval for an estimated path coefficient w does not include zero, the hypothesis that w equals zero is rejected” [98]. All of the proposed hypotheses meet this criterion, which means they are supported.

**Table 6:** Structural model results.

Hypothesis	Suggested effect	Path coefficients	t-value (bootstrap)	*p*-Value	Percentile bootstrap 95% confidence level		Support
					Lower	Upper	
H1: Perceived Social Norms→Attitude toward the Behavior	+	0.396***	4.658	0.000	0.233	0.503	Yes
H2: Perceived Social Norms→Perceived Behavioral Control	+	0.433***	7.355	0.000	0.259	0.552	Yes
H3: Attitude toward the Behavior→Intentions	+	0.368***	5.355	0.000	0.229	0.562	Yes
H4: Perceived Behavioral Control→Intentions	+	0.406***	5.429	0.000	0.318	0.549	Yes

Control variables	Path coefficients	t-value (bootstrap)	p-Value	Percentile bootstrap 95% confidence level		Support
				Lower	Upper	
Gender→Attitude toward the Behavior	0.158+	2.166	0.000	0.015	0.300	Yes
Gender→Perceived Behavioral Control	0.095ns′	1.340	0.033	−0.044	0.235	No
Sector→Attitude toward the Behavior	0.017ns′	0.173	0.863	−0.178	0.212	No
Sector→Perceived Behavioral Control	0.138ns′	1.175	0.243	−0.092	0.369	No
Size→Attitude toward the Behavior	0.095+	2.015	0.046	0.003	0.187	Yes
Size→Perceived Behavioral Control	−0.001ns′	0.011	0.992	−0.103	0.102	No
Studies→Attitude toward the Behavior	−0.119ns′	1.883	0.062	−0.244	0.005	No
Studies→Perceived Behavioral Control	−0.090ns′	1.281	0.203	−0.229	0.048	No
experience→Attitude toward the Behavior	0.052ns′	0.887	0.377	−0.063	0.167	No
experience→Perceived Behavioral Control	0.047ns′	0.741	0.460	−0.077	0.171	No

Relationships among the constructs, T-Bootstrap.

* p<0.05;

** p<0.01;

*** p<0.001; ns: not significant (based on t(4999). one-tailed test.

t(0.05; 4999) = 1.64791345; t(0.01; 4999) = 2.333843952; t(0.001; 4999) = 3.106644601.

Relationship between the dependent variable and the control variables, T-Bootstrap.

+ p<0.05;

++ p<0.01;

+++ p<0.001;

ns′ : not significant (based on t(4999). two-tailed test).

t(0.05; 4999) = 1.96043859; t(0.01; 4999) = 2.57681312; t(0.001; 4999) = 3.29247411.

In the case of the control variables, which, as mentioned above, are linked to both the Attitude toward the Behavior and the Perceived Behavioral Control, the results only support the existence of relationships between the gender of the business owner and the size of the business with the Attitude toward the Behavior (significance level of 0.05). Thus, it can be said that women and business owners who manage larger organizations have more favorable attitudes toward this type of behaviors.

In addition, to verify the validity of the model, the Stone-Geisser – Cross-validated Redundancy (Q^2^) test was performed. This test was used as a criterion to measure the predictive relevance of the dependent constructs. In the case that Q^2^>0, it indicates that the model has predictive relevance. As can be seen in Table 5, in all cases the values of Q^2^ were positive, which verifies the predictive relevancy of the model.

Finally, all of the hypotheses were found to be positive.

*Hypothesis 1*, which stated that *Perceived Social Norms have a positive influence on the Attitude toward the Behavior to undertake environmental measures*, is confirmed (*β* = 0.396; *p*<0.001).

*Hypothesis 2*, which proposed that *The Perceived Social Norms have a positive influence on the Perceived Behavioral Control to undertake environmental measures*, is accepted (*β* = 0.433; *p*<0.001).

*Hypothesis 3*, which suggested that *The Attitude toward the Behavior positively influences the Intention to undertake environmental measures,* was validated (*β* = 0.368; *p*<0.001).

*Hypothesis 4, which stated that the Perceived Behavioral Control positively influences the Intention to undertake environmental measures, was accepted (β = 0.406; p<0.001).*

## Discussion

This paper emphasizes the close relationship between businesses and the environment. In fact, businesses undoubtedly have an impact on the environment. To increase understanding about how small and medium-sized companies develop the intention to engage in actions that minimize their environmental impacts, it is necessary to analyze the determinants of this intention. Thus, it should be pointed out that in this type of businesses, innovative behavior is usually the reflection of the individual entrepreneurial intentions of the manager. In fact, the entrepreneur decisively conditions the development of small businesses, with some authors even considering them an extension of their founders [99]–[103]. The figure of the business owner and his/her perceptions are vital for carrying out actions within the company.

This research constitutes one of the first steps toward better understanding the importance of the factors in Ajzen's model [24] in explaining SME managers' intentions to carry out environmental actions. Therefore, we must emphasize that the results obtained from the analysis statistically confirm the validity of the relationships proposed in all the previously mentioned hypotheses. Perceived Social Norms have a positive influence on the Attitude toward the Behavior of undertaking environmental measures and the Perceived Behavioral Control in carrying them out. In turn, both the Perceived Behavioral Control to undertake environmental measures and the Attitude toward this Behavior have a positive influence on the Intention to implement environmental measures. Hence, the theory of planned action is validated in the context of environmental intentions, resulting in an instrument with the potential to improve the environmental management of small companies. On the other hand, it is also interesting that the attitude toward this type of behaviors is influenced by the gender of the business owner and the size of the organization he/she manages. Thus, the attitude is more favorable when the business owner is a woman or when the company has a larger size.

From a practical point of view, in a socially aware society that considers the environment important, it is likely that both the manager and the people around him/her will positively assess the performance of this type of action, thus increasing the manager's intention to carry out these actions. Therefore, beyond the legal requirements that companies may have to follow, it is necessary to encourage environmental awareness in the society, which can help managers of SMEs to engage in more proactive behavior that can improve the company's environmental performance, make it more socially responsible, and create value.

Economic, technological and knowledge resources are not sufficient if the person who manages the company does not have a positive attitude and the capacity to carry out the necessary actions. Furthermore, in small businesses concern for the environment is usually not a high priority, so that any action designed to improve this type of management is beneficial. In this sense, encouraging the environmental training of managers of SMEs can ensure that these individuals feel able to successfully develop activities that improve the environmental performance of their businesses, thus increasing their intentions to carry out such actions.

Therefore, and because the Ajzen model assumes that perceptions are learned [45], fomenting environmental awareness both in society and in managers of SMEs should be a key aspect of development and training programs. Given that one of the characteristics of the modelizations performed with the PLS technique is its predictive capacity [97], one relevant issue stemming from the present study has to do with fostering improvements in companies' environmental management. Thus, even though the study was carried out in a situation of important economic crisis, the “Attitude toward the Behavior” and the “Perceived Behavioral Control” were found to have the same weight in the entrepreneur's intention to undertake environmental initiatives in his/her company. This circumstance is important because the lack of economic resources is one of the clear determinants in business owners' consideration that they cannot carry out certain activities. Therefore, if environmental authorities or organisms want small business owners to invest money in making their companies more environmentally responsible, they can choose between two different routes. The first would be to motivate this type of measures by providing the owner with economic or technical aid; the second would be to create more awareness about this issue. Although the former would have faster results that would be easier to measure, the latter option, in spite of taking longer to produce results, would be more efficient and save the administration money in the long run. Thus, an interesting strategy would be to deploy a combination of both measures. In an initial phase, and in order to start a tendency, the first route would be used. In the following phases, the money invested in the first route would be gradually reduced in order to increase the allotment for the second route.

As a limitation of this paper, we can highlight that the firms analyzed were located on an island that depends mostly on tourism and, consequently, assesses environmental issues to a greater extent. We must also add that the data collection period coincides with a very unfavorable economic situation, which may have had an influence on the responses obtained.

Finally, future lines of investigation could extend the study to other samples (different regions, sectors, economic situations, large companies, etc.) and examine whether the intention is put into action. On the other hand, economic aspects related to investments, cost savings, etc., could be introduced into the study, along with managers' perceptions about the impact of environmental actions on customers.

## Supporting Information

Appendix S1 **Questionnaire.**
(DOCX)
