# Supplementary material for: Environmental Management in Small and Medium-Sized Companies: An Analysis from the Perspective of the Theory of Planned Behavior
Source: PLoS One. 2014 Feb 12;9(2):e88504. doi: 10.1371/journal.pone.0088504 (PMC3922869; doi:10.1371/journal.pone.0088504)
Supplement: Appendix S1 — Questionnaire. (DOCX) [file pone.0088504.s001.docx]

**Appendix S1**

Questionnaire

**Perceived Behavioral Control**

Indicate to what degree you agree with the following statements about your business capacity.

a) I am capable of initiating environmental measures in the company.

b) I can control the process of implementing environmental measures in the company.

c) I am familiar with the uses that environmental measures can offer my company

d) I know what resources are necessary to implement environmental measures.

e) If I took environmental measures, I would have a high probability of obtaining satisfactory results.

f) If I took environmental measures, I would have a high probability of success.

**Perceived Social Norms**

How much do you think improvements in environmental management are valued in your company?

a) In your immediate family

b) Among your friends

c) Among your employees

d) In your social setting

e) Among your clients

f) Among other stakeholders (administration, suppliers, etc.)

**Intention to undertake environmental measures**

Indicate your degree of agreement with the following statement:

a) I am willing to make an important economic effort to improve the environmental management of the company.

b) One of my professional objectives is to achieve a company that is better managed environmentally.

c) I will try hard to create and direct measures that contribute to improving the environment.

d) I am sure I want to take measures that improve the environmental situation of the company.

e) I will find out about subsidies and/or other types of financing that would help me to improve environmental management.

**Attitude toward the Behavior**

Rate the following statements according to your level of agreement with them.

a) Taking environmental measures would give me more advantages than disadvantages.

b) I find environmental management quite attractive.

c) If I had the opportunity and resources, I would like to take environmental measures.

d) Performing an adequate environmental management would give me great satisfaction.

e) Among other options for improvement, environmental measures are a priority for me.
